# Collection of biospecimens from parent-child dyads in a community garden-based nutrition intervention: protocol and feasibility

**DOI:** 10.1186/s40795-022-00640-6

**Published:** 2022-12-05

**Authors:** Amrik Singh Khalsa, Jonathan Burton, Michael T. Bailey, Jiangjiang Zhu, Kelly J. Kelleher, Ross M. Maltz, Brett R. Loman, Colleen K. Spees

**Affiliations:** 1 Primary Care Pediatrics, Nationwide Children’s Hospital, 700 Children’s Dr, Columbus, OH 43205 USA; 2 Center for Child Health Equity and Outcomes Research, Abigail Wexner Research Institute, Nationwide Children’s Hospital, 700 Children’s Dr, Columbus, OH 43205 United States; 3 Department of Pediatrics, College of Medicine, The Ohio State University, Columbus, OH 43210 USA; 4 Heritage College of Osteopathic Medicine, Ohio University, Dublin, OH 43016 USA; 5 Center for Microbial Pathogenesis, Nationwide Children’s Hospital, Columbus, OH 43205 USA; 6 Oral and Gastrointestinal Microbiology Research Affinity Group, Abigail Wexner Research Institute at Nationwide Children’s Hospital, Columbus, OH 43205 USA; 7 College of Education and Human Ecology, Department of Human Sciences, The Ohio State University, Columbus, OH 43210 USA; 8 James Comprehensive Cancer Center, The Ohio State University, Columbus, OH 43210 USA; 9 Division of Pediatric Gastroenterology, Hepatology and Nutrition, Nationwide Children’s Hospital, Columbus, OH 43205 USA; 10 Department of Animal Sciences, University of Illinois at Urbana-Champaign, Urbana, IL 61801 USA; 11 Division of Nutritional Sciences, University of Illinois at Urbana-Champaign, Urbana, IL 61801 USA; 12 Division of Medical Dietetics, College of Medicine, The Ohio State University, Columbus, OH 43210 USA

**Keywords:** Stool collection, Urine collection, Hair collection, Feasibility, Community-based research

## Abstract

**Background:**

Non-invasive human biospecimens, including stool, urine, and hair, are important in understanding the relationship between diet and changes in human physiologic processes that affect chronic disease outcomes. However, biospecimen collection can be difficult when collecting samples for research studies that occur away from a centralized location. We describe the protocol and feasibility in collecting stool, urine, and hair biospecimens from parents and their children at a remote location as a part of a summer community garden-based intervention.

**Methods:**

Stool, urine, and hair were collected as a part of the Summer Harvest Adventure (SHA) study, a randomized controlled, community garden-based intervention targeting children (ages 8–11 years) and their parents from low-resource neighborhoods. Biospecimens were collected from willing children and/or their parent/adult caregivers at baseline and post-intervention for evaluation of microbiome, metabolomics, and hair analyses among both intervention and control groups at a location distant from the academic laboratories conducting the analysis. The protocol used to assemble, deliver, collect, and process biospecimens are presented along with the frequencies with which specimens were successfully obtained.

**Results:**

One hundred forty six participants (73 parent-child dyads) were part of the larger SHA study and thus eligible to provide a biospecimen. A total of 126 participants, 115 participants, and 127 participants consented to provide their hair, stool and urine samples, respectively. Of the participants that consented to provide a sample, 44 children (69.8%) and 38 parents (60.3%) provided at least one hair sample, 27 children (48.2%) and 37 parents (62.7%) provided at least one stool sample, and 36 children (57.1%) and 42 parents (65.6%) provided at least one urine sample. Sample collection at the offsite location, transport, and handling at the academic center were successful and all biospecimens were deemed adequate for analyses. DNA and metabolomics yield on a subset of stool samples obtained provided excellent results in terms of an abundance of species and metabolities, as would be predicted. Urine and hair analyses are underway.

**Conclusion:**

Our work is one of the first to describe the feasibility of collecting human biospecimens, specifically stool, urine, and hair, from both parents and their children from low-resourced neighborhoods in a non-traditional garden research setting. Future work will report findings related to mechanisms between diet, microbiome, metabolites, and clinical outcomes.

**Supplementary Information:**

The online version contains supplementary material available at 10.1186/s40795-022-00640-6.

## Introduction

Biospecimens, the biological research samples taken from humans, play an important role in helping us understand the mechanistic links between dietary intake and physiological change [1–6]. Non-invasive biospecimen samples, specifically stool, urine, and hair biospecimens, are an important and growing area of research and can provide insight to internal biological processes without the need to be invasive, which can increase the willingness of participants in research studies to provide a sample. For example, in adults, the activities of the gut microbiome may mediate the relationship between dietary intake and changes in blood pressure through metabolites such as short-chain fatty acids (SCFA), which can be measured in the stool [7, 8]. Urine biospecimen samples can reflect dietary patterns that increase cardiovascular disease risk through the measure of urine trimethylamine N-oxide [9]. Hair biospecimens have been used to measure chronic stress through cortisol, and more recently have been used to estimate sugar-sweetened beverage intake through hair isotopes [10–12].

The relationships between dietary intake, intestinal and urinary microbiota, metabolites, and physiologic changes are understudied, particularly in children and families more broadly [13–15]. To fully understand the mechanistic links between dietary intake and physiologic changes, biospecimens are needed from a variety of participants, including those from minoritized racial/ethnic groups and families from low-resourced neighborhoods. However, collection of biospecimen samples from these groups has been very limited for various reasons [16, 17] including lower levels of understanding about the use of biospecimens, the need for flexible collection strategies, and a burden associated with trial participation [18–20]. Thus, efficient, feasible, and practical protocols to collect biospecimens across various settings and populations are needed.

While collection protocols have been described for research in academic centers [21, 22], often in a laboratory or clinical research space, few studies have described feasible collection protocols for studies that are physically conducted outside the academic center [22–24]. These “off-site” settings are vital in reaching a broader population, allowing recruitment of families that may not have otherwise participated in research, including from low-resourced neighborhoods or minoritized racial groups [17]. Protocols in these off-site settings require successful recruitment approaches, acceptable specimen collection methods that are feasible for families, and adequate transportation and processing of samples that maintain the integrity of the samples. Studies that seek to examine non-invasive biospecimens need to utilize valid, reproducible, and standardized methods to enhance data comparability across studies, as differences in collection methods can contribute to inter-study variability.

No study, to our knowledge, has described the feasibility of collecting non-invasive human biospecimens, specifically stool, urine, and hair, from both parents and their children in a non-traditional research setting, specifically at a garden site. Thus, we describe the research collection protocols for stool, urine, and hair biospecimens from parent-child dyads enrolled in a randomized controlled trial, Summer Harvest Adventure (SHA), a family-based, multi-component community gardening intervention.

## Materials and methods

### Subjects and overall study protocol

Summer Harvest Adventure (SHA) is a 3-year randomized controlled trial targeting children (ages 8–11 years) and their parent/adult caregivers from low-resourced neighborhoods, who were randomized to a garden-based intervention or enhanced control group. The intervention consisted of weekly evidence-based group education, produce (fruits, vegetables, and herbs) harvesting, cooking demonstrations, remote nutrition counseling informed by motivational interviewing, and e-technologies. Control families received standard USDA educational materials. Briefly, inclusion criteria included children (ages 8–11 years) and their parent/adult caregivers (herein parents), English-speaking dyads, residents of Supplemental Nutrition Assistant Program (SNAP)-eligible communities in Franklin County, Ohio (as determined by neighborhood school federal funding), and the ability to consume fruits and vegetables without concerns of any medication-nutrient interactions (e.g., warfarin). For this nested observational study, non-invasive biospecimen collection occurred in year 2 of the SHA study (2019) and was an IRB approved, optional portion of the study. Willing participants signed a supplemental consent/assent to provide stool, urine, and/or hair samples as a part of the study. Samples were collected around the time of the baseline visit (T-0) and post-intervention visits (T + 12 weeks) along with other objective and subject study measures. As this was a cross-collaboration between two academic centers, the Institutional Review Boards at all sites reviewed and approved the study.

### Biospecimen collection protocols

Stool and urine samples were collected at-home by willing children and their parents. Hair samples were collected at an offsite research location on the day of baseline and post-intervention visits, when other physical and survey measures were being obtained. Stool and urine collection kits were assembled prior to biospecimen collection. An aseptic technique during kit assembly and processing of samples was utilized to minimize laboratory-introduced contamination, including the use of gloves, lab coat, and disinfecting surfaces with 70% ethanol solution. The supplies used for the stool and urine kits, along with supplies used to collect hair samples are presented in Table 1.

**Table 1:** Contents of take-home stool and urine collection kits

Item Name	Vendor	Catalog Number	Comments/description
**Stool**			
Fisherbrand Commode Specimen Collector	Fisher Scientific	22-363-149	One per participant
Covidien Precision stool collector	Fisher Scientific	14-375-156	One per participant
**Urine**			
Parter Medical Products 90 mL sterile specimen container	Fisher Scientific	22-150-327	One per participant
**Other**			
Minigrip Labguard biohazard specimen bag	Fisher Scientific	22–311–203	Two per participant. One for stool container/gloves and one for urine container/gloves
Halyard Health purple nitrile exam gloves	Fisher Scientific	23-500-119	Two pairs (4) per participant
Freezer bag	LeaderPromos	n/a	One per participant
Gel freezer pack	LeaderPromos	n/a	One per participant
Large Carrying Bag	n/a	n/a	One bag for entire collection kit (per dyad)

#### Stool and urine kit assembly

**Fig. 1:**
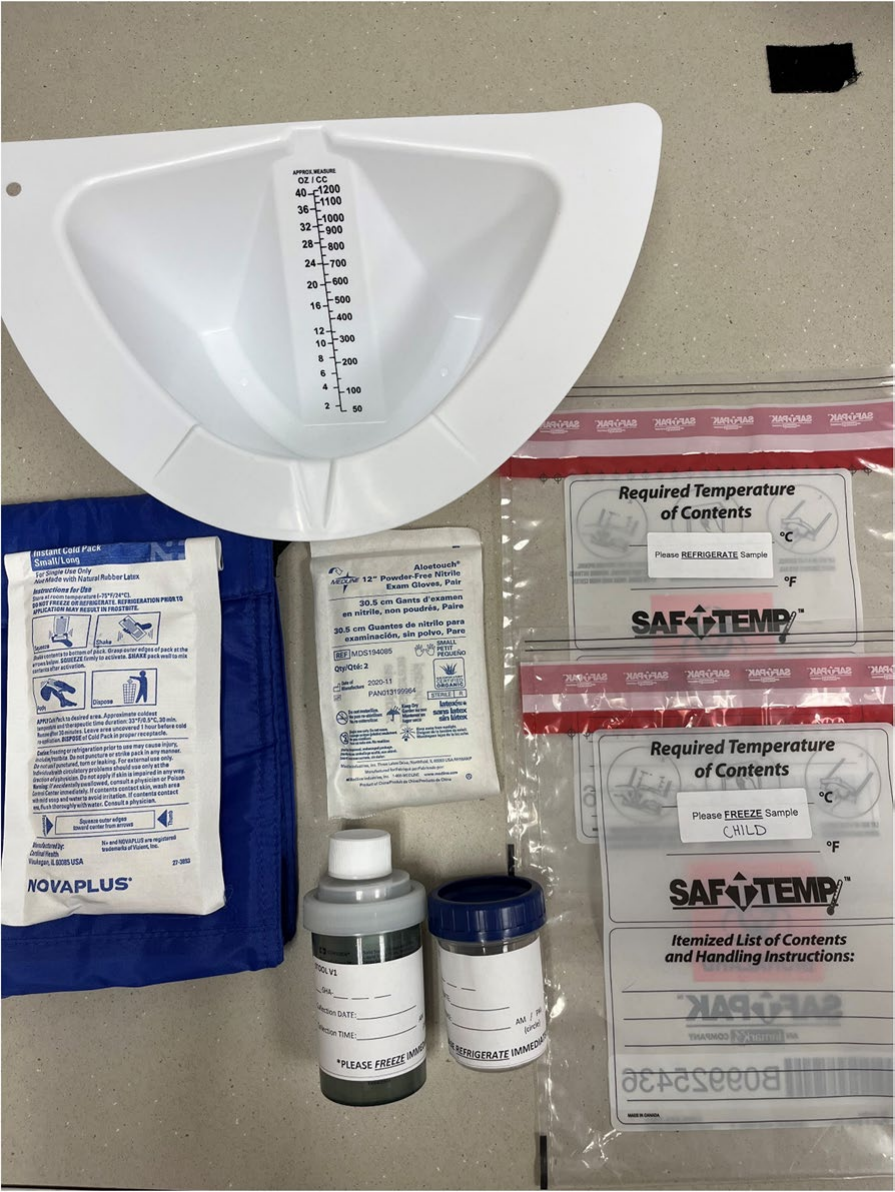
Stool and urine collection kits

The contents of each stool and urine collection kit are shown in Fig. 1. Since the stool and urine samples were to be processed for multiple purposes (e.g., microbiome composition analysis, metabolite analysis), samples were collected in containers without any preservatives (e.g., RNase later). All collection materials and containers were labelled with a study ID, including whether it was a parent or child sample, and blank lines for participants to enter the date and time the specimens were collected.

#### Pre-sample collection questionnaires

Before the distribution of stool and urine collection kits and collection of hair, a pre-sample collection questionnaire was administered to each individual that agreed to provide a sample (i.e., a separate questionnaire was administered for the parent and child if both agreed to provide a sample). Parent and child questionnaires included the following: the name, dose, and frequency of prescription medications, nutritional supplements, herbals, or botanicals; self-report of all medical diagnoses; the use and frequency of tobacco and alcohol products; date of last menstrual period (if applicable); and the presence or absence of infectious symptoms. Due to the potential effect on stool consistency and quality, the research team inquired about specific over the counter medications, including Pepto Bismol, Maalox, mineral oil, antacids, and/or Kaopectate. No participant reported taking any of these medications. Participants were told to continue any chronic medications they have been previously taking (including herbs and vitamins). Participants were also screened for any possible infectious symptoms including new diarrhea or loose/unformed stools, dysuria, increased urinary frequency, and/or current use of oral/injection antibiotics for an active infection.

#### Sample collection procedures

Hair samples were collected at the time of the baseline and post-intervention visits in a private area. For stool and urine collection, each participant was provided step-by-step instructions on how to collect samples at home. Instructions were reviewed by a trained research assistant using unused supplies as visual aids. Additionally, the instructions were detailed in a one-page handout that families took home with them (Supplemental Figs. 1 and 2). Participants were asked to produce a stool sample within three days and urine sample within two days of expected drop-off of samples to a pre-specified site during predetermined days and times.

##### Hair collection

Hair was collected using previously established protocols that were adapted for this study [11, 25]. Briefly, hair was collected from participants who had at least 1 cm of hair length. Roughly, 30 strands of hair were identified on the posterior portion of the scalp in line with top of ears. Hair scissors were wiped with alcohol prep pad before each collection and the research staff member collecting samples wore gloves. Hair was sampled using a blunt hair scissors with samples taken as close to the scalp as possible without injuring the individual. The hair sample was then taped to a sheet of paper labeled with the study ID using tape placed at least 1 cm away from the end taken from the scalp. The paper was labelled to indicate which end was from the scalp. The paper was then placed in an envelope labelled with the study ID and closed. All envelops were placed in a larger 8”x11” manila folder.

##### Stool collection

Participants were asked to produce a stool sample without urine or other fluids in the provided stool collection hat at their own convenience. Participants were asked to scoop a portion of their stool from each end and the middle into the provided collection container and close the cap of the container immediately after defecation. After appropriate hand hygiene, participants were asked to write the date and time of collection on the label outside of the container, place in biohazard bag, and immediately freeze the sample in their home freezer.

##### Urine collection

Participants were asked to collect the first void of the day, 1–2 days prior to their expected drop-off day. Participants were asked not to wipe prior to collection and void into the provided 90 mL plastic cup, closing the cap of the container after completion. After appropriate hand hygiene, participants were asked to write the date and time of collection on the label outside of the container, place in biohazard bag, and immediately place in their home refrigerator.

#### Biospecimen sample return procedures

A pre-specified community site with predetermined days and times, including a mix of day and evening times, were provided for parents to drop off the own and their child’s stool and/or urine samples. An insulated bag and several icepacks were provided to participants to transport samples from their home to the drop-off location. Participants were instructed to remove the sample(s) from their fridge/freezer immediately prior to the planned drop off and to note the time they removed the sample. Parents were reminded via email, phone, or text message up to three times (if needed) to return samples.

Upon arrival of the participant, samples were checked by trained research staff for the following: samples were brought in the provided insulated bag with icepack, were cold to touch, and appropriately labelled. A brief questionnaire was verbally administered to ensure there were no changes to their medication regimen, no new infectious symptoms, and participants adhered to the collection protocol. The date and time participants produced, placed, and removed samples from their fridge/freezer was also documented. Upon receipt from participants, samples were placed on dry ice or were immediately placed in a 4 C fridge (urine) or -20 C freezer (stool) until they were transported to the collaborating laboratory in the academic center. Transportation of samples occurred at least once a day and sometimes twice a day based on when samples were dropped-off. Samples were transported on dry ice in a closed Styrofoam container. Stool samples were immediately placed in the − 80 C freezer while urine samples were temporarily placed in a 4 C fridge for 12 h so they could be aliquoted into 15 mL conical tubes and then placed in -80 C freezer thereafter.

### Specimen processing

Hair samples were placed in a secure storage space without any access to sunlight. Stool and urine samples were processed approximately 160 (stool) and 180 (urine) days after collection to allow for the coordination of protocols, supplies, and personnel needed to process samples appropriately. Sample processing was divided between baseline samples (V1) and post-intervention samples (V2) and parent and child samples to avoid confusion. Specimens were removed from the − 80 C freezer approximately 24 h before processing and placed in a 4 C fridge in the lab to thaw. The supplies used during the initial processing of stool, urine, and hair samples are presented in Table 2 with specific details below.

**Table 2:** Biospecimen processing supplies

**Item Name**	**Vendor**	**Catalog Number**	**Comment/ Description**
Isopropanol (LCMS grade)	Fisher Scientific	A461-500	One bottle
Cryobabies Labels Laser Sheet, 1.5 – 2.0 mL Tubes	Fisher Scientific	50-998-639	One label for each planned cryovial
Thermo Scientific Nalgene Long-Term Storage 2 mL Cryogenic Tubes	Fisher Scientific	03-337-7D	One tube for each planned aliquot
**Stool**			
Sodium Butyrate (^13^C_4_99%)	Cambridge Isotope Laboratories	CLM-1256-1	One container
QIAamp DNA Stool Mini Kit	Fisher Scientific	51306	250 count
Tris Hydrochloride	Sigma	108128460010	500g
Triton X-100	Sigma	X100	
Mutanolysin (from Streptomyces globisporus ATCC21553)	Sigma	M9901-10KU	10K units
Lysozyme (from chicken egg white)	Sigma	L6876	
0.1 mm Diamter Zirconia/Silica Beads	BioSpec Products	11079101z	1lb bottle
Proteinase K	Qiagen	19131	2 mL
InhibitEX	Qiagen	19593	140 mL
Buffer AL	Qiagen	19075	264 mL
**Urine**			
Trimethylamine N-oxide (D9,98%)	Cambridge Isotope Laboratories	DLM-4779-PK	One container
**Hair**			
Cortisol Enzyme Immunoassay Kit	Salimetrics	#1-3002	
Isopropanol (HPLC grade)	Fisher Scientific	BP2632-4	
Methanol (HPLC grade)	Fisher Scientific	A452-1	
Chrome/Steel Grinding Balls	Retsch	05-368-0032	2 per sample
15 ml screw-cap polypropylene conical tube	Fisher Scientific	05-539-4	
1.5 ml round end microcentrifuge tube manufactured with reinforced plastic	Eppendorf	022363352	

#### Stool processing

The specimen container in which participants provided samples was weighed. Before placing the stool container into the biosafety hood, the containers were sprayed with 70% ethanol to sterilize the exterior surface. Once in the biosafety hood, the stool container was opened, stool consistency was assessed using the Bristol stool chart [26], and stool was homogenized with a sterilize tongue depressor. After homogenization, samples were placed in 2 mL aliquots. Three aliquots were set aside for each planned analysis with a total of 24 aliquots per individual. Additional reagents were added to those that required them (e.g., C13 Butyrate was added for SCFA analysis). All samples were weighed after completion of aliquots and placed back into the − 80 C freezer.

#### Urine processing

The 15 mL conical tubes were removed from the 4 C fridge, weighed, and sprayed with 70% ethanol to sterile the exterior surface. Urine was gently homogenized, and 1.5 mL of urine was placed in each 2 mL cryovial. Three aliquots were created for each planned analysis for a total of 12 cryovials per sample. Additional reagents were added to those that required them for future planned analyses. Cryovials were weighed and placed back in the − 80 C freezer.

### Methods to validate samples

#### Stool DNA extraction methods

Frozen stool samples were thawed and approximately 10 mg was used for DNA extraction using a QIAamp Fast DNA Stool Mini Kit (Qiagen, Hilden, Germany) using the manufacturer’s instruction with the following modifications. Contents were incubated for 45 min at 37 °C in lysozyme-mutanolysin buffer (22 mg/ml lysozyme, 0.1 U/ml mutanolysin, 20 mM TrisHCl, 2 mM EDTA, 1.2% Triton-x, pH 8.0), before homogenization for 150 s with 0.1 mm zirconia beads. Samples were then incubated at 95 °C for 5 min with InhibitEX Buffer and incubated at 70 °C for 10 min with Proteinase K and Buffer AL. Following this step, the QIAamp Fast DNA Stool Mini Kit isolation protocol was followed, beginning with the ethanol step. DNA was quantified with the Qubit 2.0 Fluorimeter (Life Technologies, Carlsbad, CA) using the dsDNA Broad Range Assay Kit.

#### Stool short chain fatty acid detection and validation analysis

Here we described briefly the validation analysis for detecting short chain fatty acid (SCFA) measurement in stools. Details including the chemicals needed, SCFA derivatization and sample preparation, and validation analysis are included in the Supplemental Appendix.

The SCFAs are generally not easy to be detected via Liquid Chromatography – Mass Spectrometry (LC-MS) system due to their volatility. Therefore, a chemical derivatization method is needed to increase their detectability as previously reported [27]. Feces were prepared with a 2:1 propanol ratio (w/w), and ^13^C_4_-sodium butyrate was spiked into these samples and served as internal standards. Then samples were frozen at -80^o^C until analysis. Before Ultrahigh Pressure Liquid Chromatography – High Resolution Mass Spectrometer (UPLC-HRMS) analysis, 60 µL of fecal sample solution was added 200 µL 50% acetonitrile for SCFAs extraction. After 2 min vortex, samples were centrifuged at 23,748 × g for 10 min and 40 µL supernatant was used for derivatization following the procedures outlined in the supplemental appendix. A Thermo Scientific Vanquish Flex UPLC coupled Q Exactive (QE) system was used to analyze derivatized SCFAs. According to the FDA guideline for bioanalytical method validation [28], the calibration curve, accuracy, precision, recovery and stability of targeted SCFAs were validated as described in our earlier study [29].

### Participant incentive

Each participant received remuneration for the samples they provided: $10 gift cards per hair and stool sample and $5 gift cards per urine sample. Thus, participants could receive up to $50 (up to $100 per dyad) if they provided all samples at baseline and post-intervention visits. An additional $5 gift card was also provided to compensate those who made an extra trip (outside pre-planned times) to deliver specimens. Compensation was provided at the time of sample drop-off.

### Statistical analysis

Means and standard deviations or frequencies and percents were used to describe demographic characteristics. Analysis of variance (ANOVA) for continuous variables and Chi-squared tests for categorical variables were used to test for differences in demographic characteristics according to whether participants consented, provided a baseline sample, or provided both a baseline and post-intervention sample. All analyses were conducted using SPSS (v28; IBM).

## Results

**Fig. 2:**
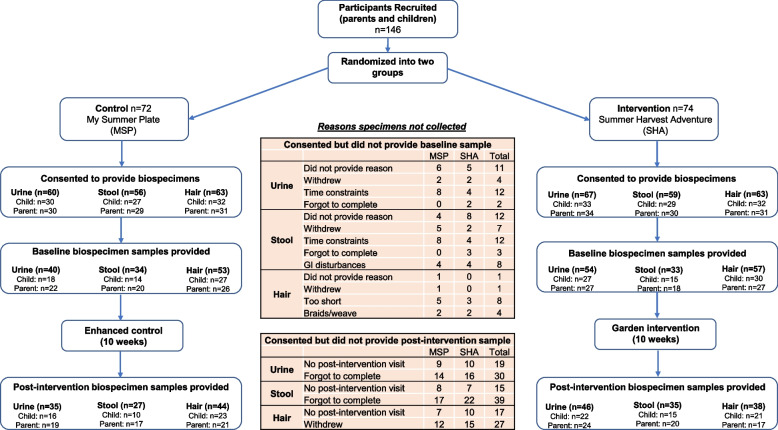
Participant flow chart

A total of 73 parent-child dyads (146 participants) agreed to participate in the SHA study in 2019 of which 36 dyads (72 participants) were randomized into the enhanced control arm and 37 dyads (74 participants) were randomized into the intervention arm. The mean age of children who participated was 8.8 years, 60.3% were female, and 54% identified as African American or mixed race (Table 3). Parents and children from each dyad were given the option to provide hair, stool and urine sample irrespective of their intervention arm (Fig. 2). A total of 126 participants (64 children, 62 parents), 115 participants (56 children, 59 parents) and 127 participants (63 children, 64 parents) consented to provide their hair, stool and urine samples, respectively (Fig. 2). Of the participants that consented to provide a hair sample, 56 children (87.5%) and 53 parents (85.5%) provided a sample at baseline and 44 children (68.8%) and 38 parents (61.3%) provided a sample at the post-intervention visit; 30 parent-child dyads provided a sample at both baseline and post-intervention visits. Of the participants that consented to provide a stool sample, 29 children (51.8%) and 38 parents (64.4%) provided a sample at baseline and 27 children (48.2%) and 37 parents (62.7%) provided a sample at the post-intervention visit; 23 parent-child dyads provided a sample at both baseline and post-intervention visits. Of the participants that consented to provide a urine sample, 45 children (71.4%) and 49 parents (76.6%) provided a sample at baseline and 36 children (57.1%) and 42 parents (65.6%) provided a sample at the post-intervention visit; 36 parent-child dyads provided a sample at both baseline and post-intervention visits. There were no significant differences between study arms in terms of children and parents who consented, provided a baseline sample, or provided both a baseline and post-intervention sample (Table 3). Demographic profiles were similar in those that provided a biospecimen sample compared to participants who did not provide a sample (Table 3).

**Table 3:** Baseline participant characteristics

**Parent/Caregiver Characteristics**	**All Particitipants**^**a**^**(*n*** **= 73)**	**Consented(*n*** **= 69)**	**Provided baseline sample**^**c**^**(*n*** **= 63)**	**Provided pre and post samples**^**c**^**(*n*** **= 45)**	***p-value***
**Age, years**	37.0 (5.7)	37.3 (5.7)	37.3 (5.7)	38 (5.7)	0.39
**Female, n (%)**	64 (87.7%)	60 (87.0%)	54 (85.7%)	36 (80.0%)	0.09
**Hispanic/Latino, n (%)**					0.09
Yes	3 (4.1%)	3 (4.3%)	3 (4.8%)	1 (2.3%)	
No	68 (93.2%)	65 (94.2%)	59 (93.6%)	43 (95.4%)	
Prefer not to answer	2 (2.7%)	1 (1.5%)	1 (1.6%)	1 (2.3%)	
**Race, n (%)**					0.18
White or Caucasian Only	31 (42.3%)	31 (44.9%)	30 (47.6%)	23 (51.1%)	
Black or African American Only	30 (41.1%)	27 (39.1%)	22 (34.9%)	14 (31.1%)	
Mixed	5 (6.8%)	5 (7.2%)	5 (7.9%)	3 (6.7%)	
Other^b^	3 (4.1%)	3 (4.3%)	3 (4.8%)	3 (6.7%)	
Don’t Know/Prefer not to answer	4 (5.5%)	3 (4.3%)	3 (4.8%)	2 (4.4%)	
**Marital Status, n (%)**					0.04
Married	35 (47.9%)	34 (49.3%)	33 (52.4%)	26 (57.8%)	
Never Married	17 (23.3%)	14 (20.3%)	13 (20.6%)	9 (20.0%)	
Divorced/Separated	10 (13.7%)	10 (14.5%)	7 (11.1%)	5 (11.1%)	
Member of an Unmarried Couple	11 (15.1%)	11 (15.9%)	10 (15.9%)	5 (11.1%)	
**Education, n (%)**					0.75
College Graduate	38 (52.1%)	37 (53.6%)	34 (54.0%)	25 (55.6%)	
Some College/Technical School	30 (41.1%)	28 (40.6%)	25 (39.7%)	17 (37.8%)	
High School Graduate/GED	5 (6.8%)	4 (5.8%)	4 (6.3%)	3 (6.7%)	
**Employment, n (%)**					0.93
Employed	53 (72.6%)	50 (72.5%)	46 (73.0%)	33 (73.3%)	
Unemployed/Unable to work	8 (11.0%)	7 (10.1%)	7 (11.1%)	5 (11.1%)	
Self Employed	8 (11.0%)	8 (11.6%)	7 (11.1%)	5 (11.1%)	
Other/Student	4 (5.5%)	4 (5.8%)	3 (4.8%)	2 (4.4%)	
**Household Income, n (%)**					0.23
≥$75,000	20 (27.4%)	20 (29.0%)	20 (31.7%)	17 (37.8%)	
$50,000 to $74,999	15 (20.5%)	13 (18.8%)	12 (19.0%)	8 (17.8%)	
$25,000 to $49,999	22 (30.1%)	21 (30.4%)	18 (28.6%)	12 (26.7%)	
$10,000 to $24,999	7 (9.6%)	7 (10.1%)	5 (7.9%)	3 (6.7%)	
≤$9,999	4 (5.5%)	3 (4.3%)	3 (4.8%)	1 (2.2%)	
Don’t Know/Prefer not to answer	5 (6.8%)	5 (7.2%)	5 (7.9%)	4 (8.9%)	
**Number of Children in Household, 18 and under, n (%)**					0.34
One	17 (23.3%)	16 (23.2%)	14 (22.2%)	7 (15.5%)	
Two	35 (47.9%)	32 (46.4%)	31 (49.2%)	26 (57.8%)	
Three or more	21 (28.8%)	21 (30.4%)	18 (28.6%)	12 (26.7%)	
**Participating Adult Relationship to Child, n (%)**					0.30
Mother	63 (86.3%)	59 (85.5%)	53 (84.1%)	35 (77.8%)	
Father	9 (12.3%)	9 (13.0%)	9 (14.3%)	9 (20.0%)	
Other	1 (1.4%)	1 (1.4%)	1 (1.6%)	1 (2.2%)	
**Participating Adult is the Nutrition Gatekeeper, n (%)**	72 (98.6%)	68 (98.6%)	62 (98.4%)	44 (97.8%)	0.89
**Intervention Arm, n (%)**					0.83
Intervention	37 (50.7%)	35 (50.7%)	33 (52.4%)	23 (51.1%)	
Control	36 (49.3%)	34 (49.3%)	30 (47.6%)	22 (48.9%)	
**Child Characteristics**	**Overall (*n* = 73)**	**Consented (*n* = 71)**	**Provided baseline sample (*n* = 68)**	**Provided pre/post samples (*n* = 46)**	***p*-value**
**Age, years**	8.8 (0.46)	8.8 (0.46)	8.8 (0.45)	8.8 (0.46)	0.51
**Female, n (%)**	44 (60.3%)	43 (60.6%)	42 (61.8%)	27 (58.7%)	0.66
**Race, n (%)**					0.10
White or Caucasian Only	27 (37.0%)	27 (38.0%)	27 (39.7%)	21 (45.7%)	
Black or African American Only	31 (42.5%)	29 (40.8%)	26 (38.2%)	15 (32.6%)	
Mixed	9 (12.3%)	9 (12.7%)	9 (13.2%)	6 (13.0%)	
Other*	3 (4.1%)	3 (4.2%)	3 (4.4%)	3 (6.5%)	
Don’t Know/Prefer not to answer	3 (4.1%)	3 (4.2%)	3 (4.4%)	1 (2.2%)	
**BMI Percentile**	65.5 (28.5)	66.5 (28.3)	65.6 (28.5)	64.4 (28.3)	0.63
**Intervention Arm, n (%)**					0.94
Intervention	37 (50.7%)	36 (50.7%)	35 (51.5%)	24 (52.2%)	
Control	36 (49.3%)	35 (49.3%)	33 (48.5%)	22 (47.8%)	

Values reported as mean and standard Deviation unless as indicated

*BMI *Body-Mass Index, *GED *General Education Diploma, *SD *Standard deviation

^a^’All participants’ includes parent/child dyads that participated in the study (both intervention or control groups). They may or may not have provided a biospecimen sample.

^b^Other includes: American Indian, Asian, Pacific Islander, South African, South East Asian

^c^These include participants who provided any sample (hair, stool, urine). Pre/Post sample provided includes those who provided the same type of sample at both time points.

Participants who consented to provide a biospecimen sample but did not return a sample were contacted. Of the 15 families who were contacted for not providing a urine sample and 22 participants who were contacted for not providing a stool sample, 11 indicated that they had time constraints, nine withdrew from this portion of the study, two stated they forgot, and three cited new gastrointestinal disturbances.

Of the participants that provided a sample, the median time from when the sample was produced to drop-off at the offsite location was 10.1 (IQR: 4.8–25.4) hours for stool and 10.4 (IQR: 8.9–78.5) hours for urine for baseline specimens and 11.2 (IQR: 6.8–29.5) hours for stool and 10.0 (IQR: 7.9–13.1) hours for urine for post-intervention specimens. All participants stored the samples as instructed at home and removed samples within an hour of dropping off their sample. The median time between when the biospecimen was produced and when specimens were frozen in -80 C freezer was 13.2 (IQR: 7.4–27.9) hours for stool and 26.7 (IQR: 24.2–28.0) hours for urine for baseline samples and 14.6 (IQR: 10.6–32.1) hours and 33.7 (IQR: 31.3–37.8) hours for post-intervention samples for stool and urine, respectively.

### Processing/DNA extraction yields

All stool and urine samples provided were found to be of sufficient quality and quantity to support additional analyses. A DNA extraction was performed on 91 samples that resulted in an average yield of 24.60 +/- 3.47 µg/ml of DNA. This DNA was submitted for 16 S rRNA gene sequencing which will be reported in future studies. In addition, SCFA analysis were conducted on 40 samples, the results of which are reported previously [30].

## Discussion

Our collaborative research group was able to demonstrate that we can successfully collect non-invasive biospecimens, specifically stool, urine, and hair samples, from both parent and child participants from low-resourced neighborhoods outside the traditional academic setting, specifically a community garden location. We developed a successful protocol to collect non-invasive biospecimen samples using established collection methods [22–24] and modified it for participant engagement and feasibility without compromising sample integrity. We believe that this protocol can be applied to other studies in similar settings outside the traditional academic research environment. While self-collection of stool is less optimal due to the potential for repeated freeze/thaw cycles, we were able to demonstrate that our protocol is practical for families while still producing valid and reliable data.

Of the 146 participants who were eligible, more than half of the parents and their children provided a biospecimen sample. Our experience suggests that increased recruitment can occur when involving demonstrations about the sample collection process coupled with clear instructions in a face-to-face manner. This is consistent with previous studies of biospecimen collection that have similarly demonstrated increased participation with face-to-face recruitment [31]. While the percent of those who consented and returned a sample was suboptimal, of the children and parents who returned a baseline sample, more than 70% provided a post-intervention sample, with a higher percent of participants providing a second stool sample compared to hair or urine sample. Most often, those that did not return a post-intervention sample also had low attendance at the gardening sessions (if they were in the intervention group) or did not return for a post-intervention visit (for both intervention and control group). It is not clear why there was a higher rate of return for stool samples although it is plausible that parents and children who opted to provide a stool sample were more engaged in the study and thus, more willing to provide a second sample. It is also plausible that these families benefited the most from the financial compensation, since the financial incentive for returning a stool sample was higher.

Previous studies have also shown that alternative protocols for collecting biospecimens outside a central collection site in an academic center is possible and feasible [22–24, 32, 33]. However, our study is the first to describe a protocol to collect multiple non-invasive biospecimens in both parents and children from low-resourced neighborhoods. In addition, we were able to show decent participation in the collection of multiple biospecimen samples in a population that has traditionally been difficult to recruit [16]. While a major downside to not collecting biospecimens in an academic center is forgoing the ability to immediately store and process samples, travel to academic centers is often not feasible for participants [19, 20]. The protocol we have outlined, we believe is a better “balance” between traditional collection methods and mail-in, at-home tests (which put the burden on the individual participant or family). We demonstrated that our protocol is convenient for families to collect samples in the comfort of their own home within a framework to ensure that sample integrity is not lost. Further, we have demonstrated that we can adequately recruit child participants as well as their parents. Child stool and urine collection methods at-home is underreported in the literature [33]. However, having both child and parent samples will allow us to test unique hypotheses in the future including concordance of microbiota and metabolites along with differences in physiologic changes that may occur due to the intervention. Understanding these relationships help us better understand the mechanisms surrounding the intergenerational risks of chronic disease, which is a growing and important area of research.

Our DNA isolation method, which is similar to that used by others, resulted in a high yield of DNA that was submitted for 16 S rRNA gene sequencing. Amplicon libraries were successfully created from every sample, and led to the generation of high quality microbiome data that will be reported in future manuscripts. Our approach has been able to mimic sample integrity to others who have used mail-in approach, however without the use of DNA stabilizers [22–24, 32].

Our protocol does have limitations. First, biospecimen collection was embedded in a larger randomized controlled trial. While biospecimen collection was successful, it was still suboptimal given lower rates of return for the post-intervention sample. Another limitation is the long delay in the initial processing of stool and urine samples which included the addition of reagents to measure metabolite decay (e.g.,13 C Butyrate). While samples were placed in at-home freezers by participants immediately and − 80 C freezer within days of production, this may decrease the stability of metabolites. Future iterations will focus on decreasing this duration of processing time. Finally, our protocol, while successful in this cohort, may not be generalizable to other populations given that this was part of a larger intervention and only focused on 8–11 year old children and their parents. Protocols may need to be adjusted for other age groups and populations.

## Conclusion

In summary, we describe a protocol for successfully collecting non-invasive biospecimens for a broad set of future analyses in parents and their children from low-resourced neighborhoods in a randomized controlled community garden-based intervention. The collection protocol was feasible and yielded samples that were suitable for analysis. This approach can be replicated for similar randomized controlled trials or observational studies.

## Supplementary Information

Additional file 1: **Supplemental Appendix.** **Supplemental Figure 1.** Home Stool sample collection instructions. **Supplemental Figure 2.** Home urine collection instructions. 

## Data Availability

The datasets used and/or analyzed during the current study are available from the corresponding author on reasonable request.
