# Supplementary material for: Collection of biospecimens from parent-child dyads in a community garden-based nutrition intervention: protocol and feasibility
Source: BMC Nutr. 2022 Dec 5;8:141. doi: 10.1186/s40795-022-00640-6 (PMC9720919; doi:10.1186/s40795-022-00640-6)
Supplement: Supplementary file 1 — Supplemental Appendix. Supplemental Figure 1. Home Stool sample collection instructions. Supplemental Figure 2. Home urine collection instructions. [file 40795_2022_640_MOESM1_ESM.docx]

**Supplemental Appendix**

**Short Chain Fatty Acid Isolation**

***a. Chemicals***

Volatile free acid mix, including acetic acid (C2), propionic acid (C3), butyric acid (C4), isobutyric acid (C4), isovaleric acid (C5), valeric acid (C5), isocaproic acid (C6, 4-methylvaleric acid), and caproic acid (C6), and 2-methylbutyric acid were purchased from Sigma-Aldrich (St. Louis, MO, USA). ^13^C_4_-sodium butyrate was purchased from Avanti polar lipids, Inc. (Alabaster, AL, USA). Pyridine, N-(3-dimethylaminopropyl)-N’-ethylcarbodiimide (EDC·HCl) and 3-Nitrophenylhydrazine hydrochloride (3NPH·HCl) were also purchased from Sigma-Aldrich. Acetonitrile and formic acid were purchased from Fisher Scientific (Pittsburgh, PA, USA). Ultrapure water was obtained through Millipore Milli-Q water purification system. 120mM EDC·HCl-6% pyridine solution and 200mM 3NPH·HCl solution were prepared with 50% acetonitrile.

***b. SCFAs derivatization and sample preparation***

The SCFAs are generally not easy to be detected via Liquid Chromatography – Mass Spectrometry (LC-MS) system due to their volatility. Therefore, a chemical derivatization method was performed to increase their detectability as previously reported (23). Briefly, for the fatty acid standard test, the mixture was diluted using 50% acetonitrile. 40 μL standard solution mixed sufficiently with 20 μL of 200 mM 3NPH·HCl solution in a 2 mL Eppendorf tube. Then 20 μL of 120 mM EDC·HCl-6% pyridine solution was added. After 2 min vortex, samples were incubated in a 40 ^o^C water bath for 30min. Samples were cooled down on ice for 1min after incubation. The derivatized samples were diluted with 0.92 mL of 10% acetonitrile before Ultrahigh Pressure Liquid Chromatography – High Resolution Mass Spectrometer (UPLC-HRMS) analysis. Feces were prepared with a 2:1 propanol ratio (w/w), and ^13^C_4_-sodium butyrate was spiked into these samples and served as internal standards. Then samples were frozen at -80^o^C until analysis. Before UPLC-HRMS analysis, 60 µL of fecal sample solution was added 200 µL 50% acetonitrile for SCFAs extraction. After 2min vortex, samples were centrifuged at 23748 × g for 10min and 40 µL supernatant was used for derivatization following the same procedures described above.

***c. UPLC-HRSM analysis***

A Thermo Scientific Vanquish Flex UPLC coupled Q Exactive (QE) system was used to analyze derivatized SCFAs. The QE was coupled to a universal ion source with Ion Max heated electrospray ionization (HESI-II) probe. The PRM mode was used for qualitative determination of SCFAs, while t-SIM mode was used for quantitative analysis. The mass spectra were recorded in negative ion mode. The QE mass parameters were as follows: sheath gas flow rate 10 mL/min, spray voltage 4000V, capillary temperature 320 ^o^C, resolution 70000 FWHM, Automatic gain control target 5 x 10^4^ (t-SIM), 2 x 10^5^ (PRM), and 1 x 10^6^ (tune method). Separation was performed with Waters CSH C18 (2.1x100mm, 1.7µm) column. The samples were introduced into LC using an auto-sampler (5µL, maintained at 6 ^o^C) in a two-mixture mobile phase: mobile phase A was composed of water (0.1% formic acid), and B was acetonitrile (0.1% formic acid). The initial condition of 15% B held for 2 min and then linearly ramped to 55% B by 9 min, then to 100% B by 0.1min and held for 1 min. The final value was then decreased linearly to 15% B by 0.1 min and held for 4 min. The mobile phase flow rate was held constant at 0.35mL min^-1^. According to the FDA guideline for bioanalytical method validation (24), the calibration curve, accuracy, precision, recovery and stability of targeted SCFAs were validated as described in our earlier study (25).

**Supplemental Figure 1: Home Stool Sample Collection Instructions**

**BEFORE YOU BEGIN – PLEASE READ:**

**Please DO NOT collect a stool sample if you are taking any of the following medications: Pepto Bismol, Maalox, mineral oil, antacids, and Kaopectate.**


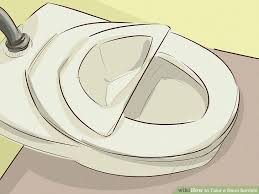
**Please DO NOT collect a stool sample if you actively have diarrhea, any loose/unformed stools, or are being treated for an infection with antibiotics.**

1. Empty your bladder before collecting stool. Stool sample should be free of urine, water, and toilet paper.
2.
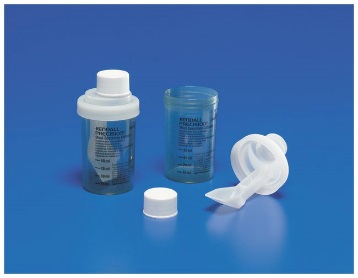
Life up the toilet seat and **place the plastic hat (provided) over the bowl**, and then lower the toilet seat again.
3. After collecting your stool sample in the hat, put on the gloves provided to prepare transferring the stool into the plastic container (provided).


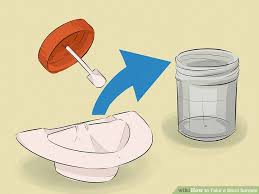


1. Open the top lid slowly so that the plastic shovel-like tool is out.
2. Use the provided shovel-like tool to scoop a small amount of stool.
3. Transfer the stool sample into the plastic container using the shovel-like tool.
4. Repeat this process until the sample fills **three-fourths** of the container. **Try to take some stool from each end and from the middle.**
5. You can use tissues or paper towel to wipe the sides as needed.
6. Screw the shovel-like tool back on top. Make sure it’s secure. Ensure the plastic cap is also on. Remove gloves and wash hands thoroughly.
7.
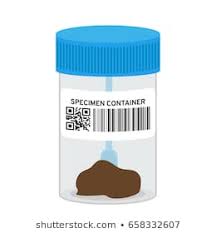
**Fill out the label with the date AND time of collection. Make sure the container with a ‘P’ is the parent sample and ‘C’ is the child sample.**
8. Place stool sample in provided plastic bag and **store in freezer**. Please freeze immediately after collection.

**Bring the sample to your next visit. Place sample in cooler with icepack (provided) for travel.**

**If you have any questions, please call [Name of Research Assistant] at [Study Phone #]**

**Supplemental Figure 2: Home Urine Collection Instructions**

**BEFORE YOU BEGIN – PLEASE READ THE FOLLOWING INSTRUCTIONS:**

**Please DO NOT** collect urine sample if you are taking antibiotics.

**Please DO NOT** collect urine sample if you currently have signs/symptoms of an infection (pain or burning with urination, increased frequency).

1.
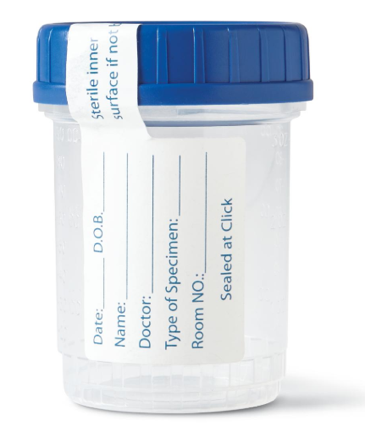
**We are asking you to collect a urine sample when you first wake-up for the day (the first time you urinate for the day).**
2. Wash your hands before collecting urine sample. You will need the blue plastic urine cup.
3. Collect your sample by urinating directly into the cup. **The sample should be free of water or stool.**
4. Screw the lid on tight, make sure it’s secure. Wash your hands thoroughly and dry.
5. **Fill out the label with the date AND time of collection.**

**Make sure the container with a ‘P’ is the parent sample and ‘C’ is the child sample.**

1. Place urine cup in provided plastic bag and store in your fridge. **Please store in the fridge immediately after collection.**

Bring the sample with you at your next visit. **Place sample in cooler with icepack (provided) for travel when dropping off.**

Note: It can be placed in the same cooler as the stool sample.

**If you have any questions, please call [Name of Research Assistant] at [Study Phone #]**
